# Rapid Phenotypic Convergence towards Collateral Sensitivity in Clinical Isolates of Pseudomonas aeruginosa Presenting Different Genomic Backgrounds

**DOI:** 10.1128/spectrum.02276-22

**Published:** 2022-12-19

**Authors:** Sara Hernando-Amado, Carla López-Causapé, Pablo Laborda, Fernando Sanz-García, Antonio Oliver, José Luis Martínez

**Affiliations:** a Centro Nacional de Biotecnología, CSIC, Madrid, Spain; b Servicio de Microbiología, Hospital Universitario Son Espases, Instituto de Investigación Sanitaria Illes Balears, CIBERINFEC, Palma de Mallorca, Spain; c Departamento de Microbiología, Medicina Preventiva y Salud Pública, Universidad de Zaragoza, Zaragoza, Spain; Emory University School of Medicine

**Keywords:** *Pseudomonas aeruginosa*, clinical isolates, antibiotic resistance, collateral sensitivity, evolutionary robustness, quinolones, drug resistance evolution

## Abstract

Collateral sensitivity (CS) is an evolutionary trade-off by which acquisition of resistance to an antibiotic leads to increased susceptibility to another. This Achilles’ heel of antibiotic resistance could be exploited to design evolution-based strategies for treating bacterial infections. To date, most studies in the field have focused on the identification of CS patterns in model strains. However, one of the main requirements for the clinical application of this trade-off is that it must be robust and has to emerge in different genomic backgrounds, including preexisting drug-resistant isolates, since infections are frequently caused by pathogens already resistant to antibiotics. Here, we report the first analysis of CS robustness in clinical strains of Pseudomonas aeruginosa presenting different *ab initio* mutational resistomes. We identified a robust CS pattern associated with short-term evolution in the presence of ciprofloxacin of clinical P. aeruginosa isolates, including representatives of high-risk epidemic clones belonging to sequence type (ST) 111, ST175, and ST244. We observed the acquisition of different ciprofloxacin resistance mutations in strains presenting varied STs and different preexisting mutational resistomes. Importantly, despite these genetic differences, the use of ciprofloxacin led to a robust CS to aztreonam and tobramycin. In addition, we describe the possible application of this evolutionary trade-off to drive P. aeruginosa infections to extinction by using the combination of ciprofloxacin-tobramycin or ciprofloxacin-aztreonam. Our results support the notion that the identification of robust patterns of CS may establish the basis for developing evolution-informed treatment strategies to tackle bacterial infections, including those due to antibiotic-resistant pathogens.

**IMPORTANCE** Collateral sensitivity (CS) is a trade-off of antibiotic resistance evolution that could be exploited to design strategies for treating bacterial infections. Clinical application of CS requires it to robustly emerge in different genomic backgrounds. In this study, we performed an analysis to identify robust patterns of CS associated with the use of ciprofloxacin in clinical isolates of P. aeruginosa presenting different mutational resistomes and including high-risk epidemic clones (ST111, ST175, and ST244). We demonstrate the robustness of CS to tobramycin and aztreonam and the potential application of this evolutionary observation to drive P. aeruginosa infections to extinction. Our results support the notion that the identification of robust CS patterns may establish the basis for developing evolutionary strategies to tackle bacterial infections, including those due to antibiotic-resistant pathogens.

## INTRODUCTION

Infections caused by antibiotic-resistant bacterial pathogens constitute an increasing threat to human health. Among these pathogens, Pseudomonas aeruginosa stands out (1, 2). It is an opportunistic pathogen with a high capacity to cause nosocomial infections, as well as chronic infections, in patients suffering from cystic fibrosis or chronic obstructive pulmonary disease (3–5). In addition to its intrinsic low susceptibility to antibiotics (6, 7), P. aeruginosa can increase its level of resistance through the acquisition of mutational changes that lead to a reduced drug uptake, an overproduction of either efflux pumps or antibiotic-inactivating enzymes, or a modification of the drug target, among other mechanisms, resulting in a specific mutational resistome (8, 9). It is also worth mentioning that there are several concerning epidemic clones of P. aeruginosa, the denominated high-risk clones, which exhibit multidrug and extensive drug resistance, being a growing threat in hospitals worldwide (10). Consequently, this microorganism has been included in the groups of bacteria causing the greatest concern regarding antibiotic resistance (1, 2). In this context, novel therapeutic strategies are needed to improve the efficacy of available antibiotics (11) and to limit the selection of novel resistance mechanisms. For that purpose, an Achilles’ heel of antibiotic resistance that could be exploited to tackle bacterial infections is collateral sensitivity (CS).

CS, a term coined in the 1950s (12), is a trade-off antibiotic resistance evolution whereby acquisition of resistance to an antibiotic leads to an increased susceptibility to another (13–15). Several adaptive laboratory evolution (ALE) works have focused on the study of the combination (16–18) or the alternation (19–22) of drug pairs using susceptible model strains, in order to unveil CS patterns. Although some robust cases of this phenomenon have been described for model strains (20, 23, 24), this is not a common trait. Indeed, different patterns of CS in replicate populations of a single genomic background subjected to evolution in the presence of only one antibiotic have been observed (14, 25–27). This frustrates the applicability of this evolutionary trade-off even in a utopian situation in which the infections were caused by only a single susceptible model strain. Moreover, CS patterns are rarely found to be conserved when different isolates of the same species are compared (28). The reason is that pleiotropic and epistatic phenomena may shape the fitness costs associated with antibiotic resistance mutations, reducing the possible genetic variations that can be selected in a specific genomic background (29–32), as well as their associated trade-offs.

To further complicate the exploitation of CS, preexisting antibiotic-resistant mutants—rather than model, susceptible, strains—are the ones frequently encountered in clinics. In the case of P. aeruginosa, infections in cystic fibrosis patients give rise to the selection of different mutants, presenting different morphotypes and with distinct antibiotic susceptibility profiles, due to the exposure to several and extended antimicrobial therapies (9). These mutants, which may present a different mutational resistome (8, 33) and, consequently, different CS patterns if they were treated with a specific drug, coexist in the lungs of infected patients. Moreover, it has been described that the loss of function of a single gene, *lasR*, which regulates the quorum sensing response in P. aeruginosa (34) and whose inactivation is frequently selected in infected cystic fibrosis patients (35, 36), can modify the evolution of both antibiotic resistance and CS in the P. aeruginosa PA14 model strain (29). This lack of conservation of CS found when different strains are analyzed makes this phenotype rather unpredictable and limits its clinical exploitation to those situations in which a robust and predictable pattern of CS emerges when a drug is used (37). This is the reason why the identification of robust CS networks associated with drugs commonly used to treat infections caused by P. aeruginosa, such as quinolones, is of utmost interest (38).

Ciprofloxacin is a quinolone extensively used to treat infections caused by P. aeruginosa (39, 40). Resistance to this antibiotic in this bacterium may occur by the acquisition of mutations in either of the ciprofloxacin target-encoding genes *parCE* (encoding DNA topoisomerase IV) and *gyrAB* (encoding DNA gyrase) (41–45) and by the acquisition of mutations in genes coding for negative regulators of the expression of efflux pumps that extrude ciprofloxacin (i.e., MexCD-OprJ, MexEF-OprN, and MexXY-OprM) (39, 43, 46–48), such as *nfxB*, *mexZ*, and *mexS* (20, 48, 49). Importantly, ciprofloxacin-resistant clinical isolates of P. aeruginosa have been described to display CS to aminoglycosides (20), a family of drugs frequently used to treat P. aeruginosa infections (50, 51).

Phenotypic convergence toward CS may be the result of parallel evolution, in which the same genetic event is selected regardless of the genomic background (37), or it may result from the acquisition of different genetic modifications (52, 53). Accordingly, we recently described that different ciprofloxacin resistance mutations of clinical relevance (39, 41, 45, 48) are selected in different mutational backgrounds of P. aeruginosa PA14 when they are subjected to short-term ALE in the presence of ciprofloxacin and that they lead to a conserved CS to tobramycin and aztreonam (38). This provides an example of phenotypic convergence in the absence of parallel evolution that could be exploited to tackle antibiotic resistance. Additionally, we observed that, in agreement with a previous work performed in the PA14 model strain (16), the combinations ciprofloxacin-tobramycin and ciprofloxacin-aztreonam are extremely effective in driving preexisting antibiotic-resistant mutants of PA14 to extinction. However, it needed to be confirmed if the use of ciprofloxacin during ALE of clinical strains of P. aeruginosa, presenting different genomic backgrounds and different mutational resistomes, could also lead to a robust CS to tobramycin and aztreonam, an essential requirement for the extrapolation of the previous findings to clinical practice.

In this work, we tackled the above question by using 25 clinical isolates of P. aeruginosa presenting different sequence types (STs) and mutational resistomes and, therefore, distinct antibiotic susceptibility profiles. In addition, we included recognized high-risk epidemic clones belonging to ST111, ST175, and ST244 (10, 54). We observed that ciprofloxacin selects a robust pattern of CS to aztreonam and tobramycin and that this phenotype may be exploited to drive antibiotic-resistant clinical strains of P. aeruginosa to extinction. All of this supports the idea that the identification of robust patterns of CS can be the basis for the rational development of evolution-based strategies for treating P. aeruginosa infections.

## RESULTS AND DISCUSSION

### Evolution of ciprofloxacin resistance after short-term evolution in the presence of ciprofloxacin.

**FIG 1:**
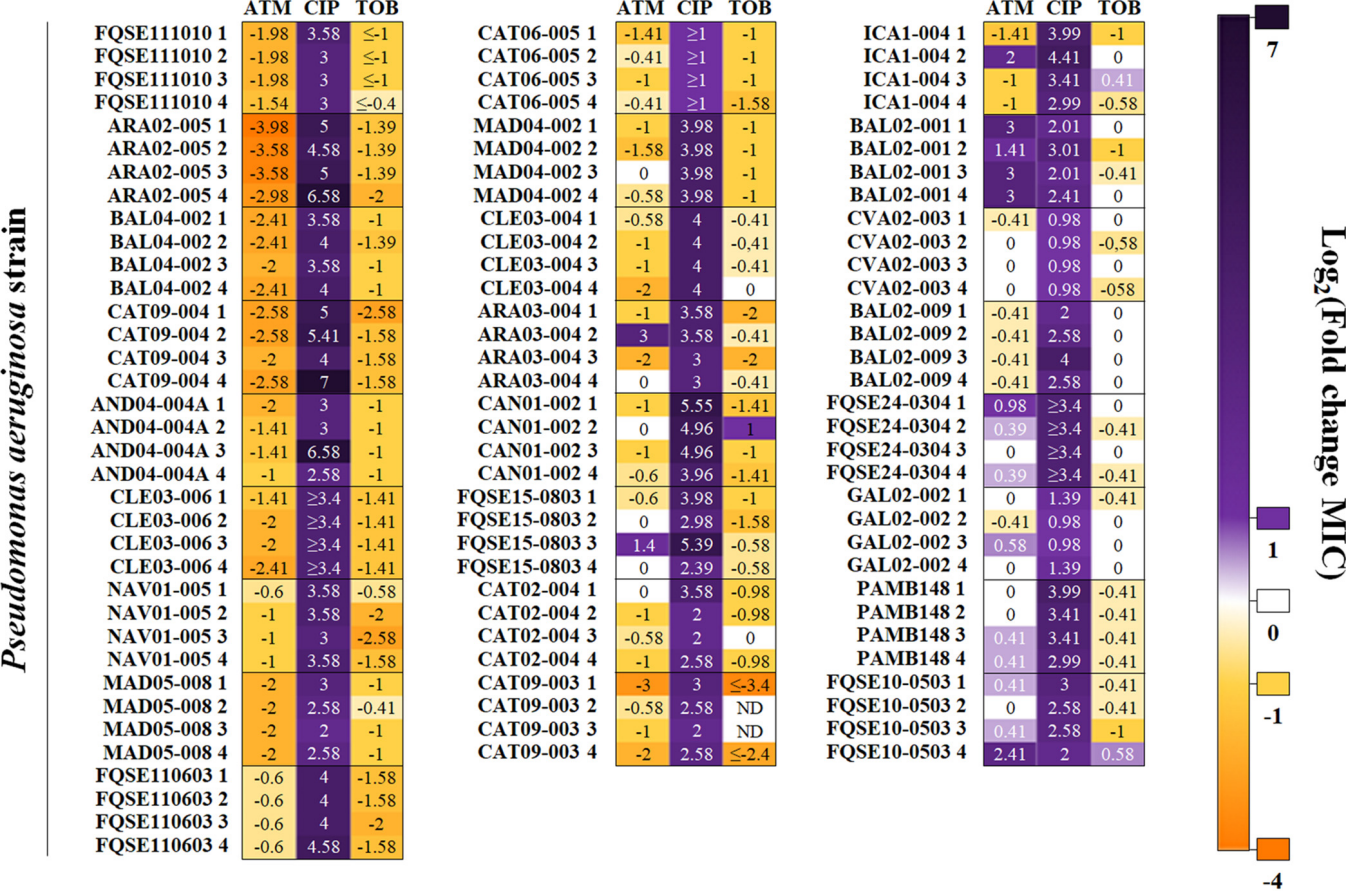
Robustness of collateral sensitivity to tobramycin and aztreonam in clinical isolates of P. aeruginosa subjected to short-term ALE on ciprofloxacin. Resistance to ciprofloxacin and CS to aztreonam or tobramycin were analyzed in 25 clinical isolates, 4 replicate populations each (100 populations)—indicated as 1, 2, 3, or 4—subjected to ALE in the presence of ciprofloxacin for 3 days. Since control populations evolved in the absence of antibiotic (100 populations) may present subtle changes (below or above a 2-fold increase or decrease) in their susceptibility to antibiotics with respect to the MIC of parental strains, only changes in MICs above or below a 2-fold increase or decrease were considered biologically relevant to classify a population as resistant (purple) or susceptible (yellow). MIC values (in micrograms per milliliter) of all the evolved populations are included in Table S1. MIC values (in micrograms per milliliter) of control populations evolved in the absence of drugs are included in Table S2. Log_2_ fold change MIC values are indicated. When evolved populations contained different subpopulations presenting diverse MICs, the MIC with a greater change from the parental strain is represented. Each column contains the log_2_ fold change MIC for each antibiotic in every replicate population. TOB, tobramycin; CIP, ciprofloxacin; ATM, aztreonam; ND, fold change could not be calculated because MICs were above the limit of detection.

Four biological replicates of 25 clinical isolates (presenting varied STs, mutational resistomes, and antibiotic susceptibility profiles) from different Spanish hospitals (Table 1) were subjected to ALE in the presence or absence (control populations) of ciprofloxacin for 3 days, with daily sequential dilutions in test tubes (a total of 200 populations). As expected, an increase of ciprofloxacin MICs, ranging from 2-fold up to 127-fold, was observed in all the populations evolved in the presence of ciprofloxacin (Fig. 1; see also Table S1 in the supplemental material). Some evolved populations presented a mixture of subpopulations with different antibiotic resistance levels. In these cases, MICs of both subpopulations, as well as the abundance of each subpopulation, were determined (Table S1). Comparisons between the MICs of ciprofloxacin before and after ALE using the nonparametric Wilcoxon test (see Materials and Methods) indicated that the differences were significant in all strains. When the fold changes of ciprofloxacin MICs after ALE assays in the presence of ciprofloxacin (Table S1) were compared with the ones of control populations evolved in the absence of drugs (Table S2), significant differences were found in all cases (*P* < 0.05), using the *t* test to compare their respective log_2_ fold change values. Control populations did not generally present an increase of MIC to ciprofloxacin with respect to the MIC of parental strains, and when these changes occurred, they were minor (below or above a 2-fold increase or decrease). No statistically significant increases in ciprofloxacin MIC were observed in any of the control strains when considering the change measured as log_2_ fold change (*P* < 0.05), with the only exception of CAN01-002. This feature supports the idea that the changes in MICs (above or below a 2-fold increase or decrease) of the populations evolved in the presence of ciprofloxacin were due to antibiotic selection, not to a nonspecific adaptation to the growth medium, and that, in agreement with previous analyses (38, 55), this threshold supports the biological significance of these findings.

**TABLE 1:** Clinical isolates used in this study^*a*^

Isolate	Sample origin	ST	Hypermutator	MIC (mg/L)			Acquired resistome	Mutational resistome
				CIP	ATM	TOB		
CAN01-002	Sputum	111	No	0.032	0.38	2		*mexB* (Q319X), *mexY* (G530S), *mexZ* (Q140K), *mexT* (G276D), *oprD* (nt174Δ11), *mexS* (nt300IS)
CAT06-005	Sputum	175	No	16	4	48	*aadB*	*oprM* (T198P), *oprD* (Q142X), *mexZ* (G195D), *gyrA* (T83I, D87N), *ampR* (G154R), *parC* (S87W), *armZ* (V266M)
MAD04-002	Sputum	242	No	0.19	6	2		*parC* (K726R)
AND04-004A	Endotracheal aspirate	244	No	0.125	4	1		
CAT02-004	Blood	244	No	0.5	12	0.75		*mexR* (nt266ins1), *dacB* (A340G)
CAT09-004	Blood	244	No	0.094	3	1.5		
CAT09-003	Sputum	253	No	0.25	6	≥256	*aacA4*	*mexZ* (H51D)
ARA02-005	Blood	267	No	0.125	3	1		*oprN* (R151L), *armZ* (R320C)
FQSE10-0503	CF sputum	274	No	0.5	3	4		*mexY* (V875M), *mexZ* (IS)
FQSE15-0803	CF sputum	274	Yes, *mutS* (nt814Δ4)	0.38	0.38	3		*mexA* (L338P), *mexZ* (A144V)
PAMB148	Blood	274	No	0.094	96	4		*mexY* (V875M), *ampD* (P41L)
CLE03-004	Endotracheal aspirate	381	No	0.125	3	1		*mexZ* (nt386Δ1)
CVA02-003	Sputum	640	No	0.38	4	48		*gyrB* (S466F), *mexA* (E53X), *parS* (S33R), *mexY* (T472P), *mexZ* (nt491Δ10), *fusA1* (R680C)
ICA01-004	Sputum	698	No	0.094	4	1.5		*oprD* (D43N), *mexE* (V156A), *ampD* (E162Q), *armZ* (V266M), *mexY* (D428N)
FQSE11-0603	CF sputum	701	No	0.125	0.38	3		*mexB* (nt775Δ1), *mexY* (N709H, A586T), *mexX* (A38P), *oprN* (R363H), *ampDh2* (P116S)
FQSE11-1010	CF sputum	701	No	0.5	0.75	≥256	*aadB, aacA4*	*gyrB* (R138L), *mexY* (N709H, A586T), *mexX* (A38P), *oprN* (R363H), *gyrA* (Y267N), *nfxB* (E75K), *ampDh2* (P116S), *mpl* (Q248X), *mexA* (nt45Δ1)
ARA03-004	Sputum	845	No	1	8	4		*gyrA* (D87N), *ampD* (Q131X), *mexT* (G246S), *mexB* (nt54Δ1), *mexZ* (nt302Δ9), *dacB* (nt781Δ1)
FQSE24-0304	CF sputum	1089	No	3	0.38	4		*gyrB* (S466F), *mexA* (L338P), *oprM* (E456G), *oprD* (Q67*), *mexY* (Y355H), *mexZ* (A194P), *galU* (P123L), *fusA1* (K430E), *pmrB* (R287Q), *mexF* (nt1074ins9)
CLE03-006	Blood	1337	No	3	4	4		*parR* (aa214Δ1), *gyrA* (D87N), *ampD* (D59E)
BAL02-001	Sputum	1619	No	0.047	6	2		
NAV01-005	Sputum	1637	No	0.5	0.38	12		*gyrB* (P749S), *mexB* (P190L), *oprD* (nt742Δ1), *mexZ* (R104W), *galU* (F248S), *fusA1* (Y552C), *mexD* (L1027V), *mexC* (V367A), *pmrB* (A173T), *armZ* (A262S)
MAD05-008	Sputum	1717	No	0.5	8	4		*parS* (V152A), *mexY* (T238I)
BAL04-002	Blood	1816	No	0.125	4	1		*mexA* (K86E), *ampR* (G295R), *ampC* (A278G), *parE* (E215Q)
BAL02-009	Sputum	2124	No	1	2	0.5		*ampDh3* (P55S), *mexY* (R251H), *mexX* (A38T), *mexZ* (G68D), *mexS* (V104A), *ampR* (A16V), *ampD* (T47I), *nfxB* (R163Q)
GAL02-002	Sputum	3342	No	0.38	8	8		*mexZ* (Y204X), *galU* (C242X), *mexS* (G76S), *oprJ* (D303V), *pmrB* (A467V), *mexD* (nt3068ins9)

a CF, cystic fibrosis; CIP, ciprofloxacin; ATM, aztreonam; TOB, tobramycin; ins, insertion; IS, insertion sequence; X, stop codon. Δ, deletion.

As mentioned, the populations subjected to ALE in the presence of ciprofloxacin presented a statistically significant increase of ciprofloxacin MIC (*P* < 0.05) (Fig. 1; Table S1). It is worth noting that the greatest increase of ciprofloxacin resistance occurred in isolates CAT09-004, ARA02-005, AND04-004A, CAN01-002, FQSE15-0803, FQSE11-0603, and ICA01-004, while this increase was subtler in other isolates, indicating that some genomic backgrounds are more prone to increase their level of resistance than others in the presence of ciprofloxacin. Overall, the clones presenting lower fold changes of ciprofloxacin MIC did not present a higher initial MIC to this antibiotic than those presenting a greater fold change in their level of resistance to ciprofloxacin. Interestingly, the isolates that suffered an important increase of ciprofloxacin MIC did not originally present mutations in *gyrAB*, which encodes the target of quinolones (41–45), in *mexS*, which encodes the regulator of the expression of *mexEF-oprN*, or in *nfxB*, which encodes the regulator of the expression of *mexCD-oprJ* (39, 43, 46–48), except isolate CAN01-002, which presented mutations in *nfxB*. In contrast, some of the isolates that suffered a low increase of ciprofloxacin MIC originally presented mutations in the mentioned genes—*gyrA* (CAT06-005), *gyrB* (CVA02-003), or *mexS* (GAL02-002)—suggesting that evolution toward ciprofloxacin resistance is contingent on the preexisting quinolone mutational resistome. In fact, in a previous work we observed that ciprofloxacin selects for either *gyrAB*, *mexS*, or *nfxB* ciprofloxacin resistance mutations in different preexisting resistant mutants of P. aeruginosa PA14, depending on the mutational background (38). The question here was whether the observed differences in the increase of ciprofloxacin resistance in the different clinical isolates after ALE assays were due to their distinct original genomic backgrounds and mutational resistomes, taking into account that some isolates originally presented well-known ciprofloxacin resistance mutations (Table 1), and whether said differences could also shape the evolution of CS.

### Conservation of collateral sensitivity after short-term evolution in the presence of ciprofloxacin.

In order to analyze the possible evolutionary conservation of CS to tobramycin and aztreonam associated with the short-term use of ciprofloxacin in the different clinical isolates of P. aeruginosa, MICs were determined for each final population and its parental strain (Tables S1 and S2). Interestingly, CS to tobramycin or aztreonam was observed in 18 or 16 out of 25 evolved genomic backgrounds, respectively (Fig. 1; Table S1), in a total of 63 out of 100 populations; including clinical isolates belonging to recognized high-risk epidemic clones (ST111, CAN01-002; ST244, CAT09-004, AND04-004A, and CAT02-004; and ST175, CAT06-005) (10, 54). A decrease of tobramycin MIC, ranging from 2-fold up to 10.6-fold, and a decrease of aztreonam MIC, ranging from 2-fold up to 15.8-fold, were observed in populations evolved in the presence of ciprofloxacin (Table S1). Reductions in tobramycin MICs were statistically significant (*P* < 0.05) in all cases but CAN01-002 and CAT09-003. Likewise, reductions observed in aztreonam MICs were statistically significant (*P* < 0.05) in all cases but ARA03-004 and ICA01-004. Interestingly, clinical strains presenting an important decrease of tobramycin or aztreonam MIC, such as CAT09-004 or ARA02-005 and CAT09-004, respectively, did not originally present mutations in *gyrAB*, *mexS*, or *nfxB*, mutations that, in addition to conferring quinolone resistance, also led to CS to tobramycin and aztreonam in preexisting resistant mutants of P. aeruginosa PA14 (38), or in other ciprofloxacin resistance genes, such as *parCE*. However, a decline of tobramycin or aztreonam MICs also occurred in clinical strains originally presenting mutations known to be involved in quinolone resistance, such as *gyrA* (ARA03-004, CAT06-005, and CLE03-006), *nfxB* (FQSE11-1010), or *mexS* (CAN01-002) (Table 1), suggesting that even clinical strains presenting ciprofloxacin resistance mutations may evolve toward CS to tobramycin and/or aztreonam when ciprofloxacin is applied. Therefore, the question here was the extent to which the original genomic background as well as the quinolone mutational resistome might shape the novel ciprofloxacin resistance genetic variations acquired and, eventually, CS to aztreonam and tobramycin.

### Ciprofloxacin resistance mutations associated with collateral sensitivity in different antibiotic-resistant clinical strains of P. aeruginosa.

**FIG 2:**
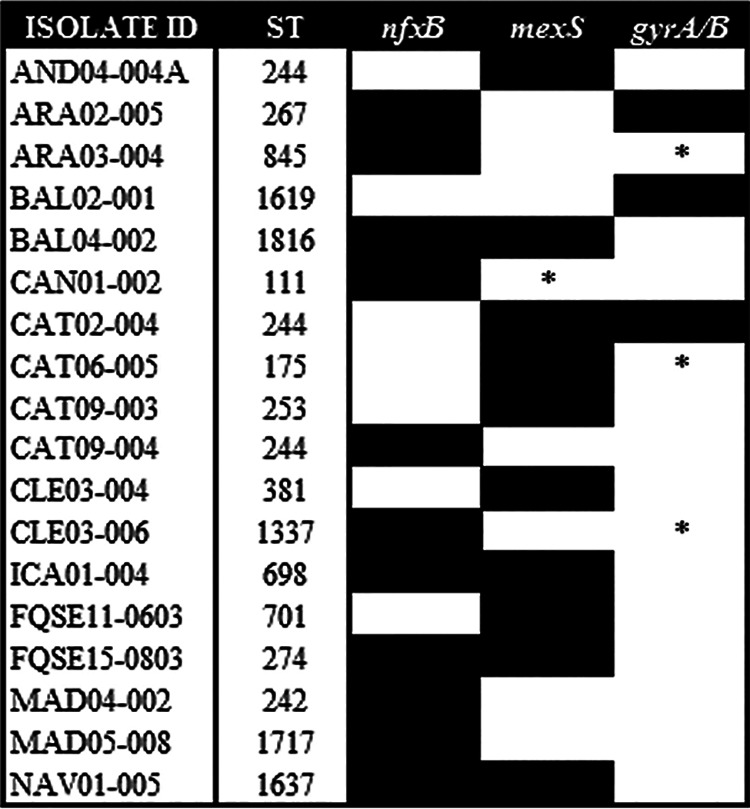
Diagram showing the genomic background dependence of the acquisition of ciprofloxacin resistance mutations leading to collateral sensitivity in clinical strains of P. aeruginosa. Classical mutations regularly implicated in ciprofloxacin resistance in clinical settings, within *gyrAB*, *nfxB*, and *mexS* genes, were acquired (black boxes) during ALE in the presence of ciprofloxacin for 3 days in populations belonging to 18 different clinical isolates. For *gyrAB*, we included only cases where genetic variations are present within the quinolone resistance-determining regions. Original mutated genes are indicated with an asterisk (Table 1). We identified 33, 36, 3, and 1 genetic variant of *mexS*, *nfxB*, *gyrA*, and *gyrB*, respectively. Genomic background and the presence of preexisting quinolone resistance mutations in *gyrAB*, *nfxB*, and *mexS* restrict early steps of ciprofloxacin resistance evolution (Table S3).

In order to delve into the genetic causes responsible for the acquisition of ciprofloxacin resistance and the robust CS pattern observed in 63 out of 100 populations subjected to short-term ALE in the presence of ciprofloxacin, these populations were subjected to whole-genome sequencing. The mutational resistomes of the ciprofloxacin-resistant populations were compared to the ones of their respective parental strains in order to determine new genetic events acquired during evolution. All detected genetic variants were located just within 4 different genes: *mexS* and *nfxB*, encoding regulators of the expression of *mexEF-oprN* and *mexCD-oprJ*, respectively, which may lead to overexpression of the efflux pumps encoded by these genes (39, 43, 46–48); and *gyrAB*, encoding the quinolones’ target (41–45) (Fig. 2; Table S3). In fact, mutations in these genes have been frequently reported for P. aeruginosa isolates from chronically infected cystic fibrosis patients treated with ciprofloxacin (20, 48, 49).

Importantly, we observed that the acquisition of genetic variations in these genes was dependent, to some extent, on the genomic background (Fig. 2; Table S3): *mexS* variants were acquired in 10 different clinical isolates (NAV01-005, BAL04-002, FQSE11-0603, AND04-004A, FQSE15-0803, CAT02-004, CAT09-003, CLE03-004, ICA01-004, and CAT06-005), *nfxB* variants were acquired in 11 different clinical isolates (NAV01-005, ARA03-004, ARA02-005, BAL04-002, CAN01-002, CAT09-004, ICA01-004, MAD04-002, CLE03-006, FQSE15-0803, and MAD05-008), *gyrA* variants were acquired in 3 different clinical isolates (BAL02-001, CAT02-004, and CAT09-004) and a *gyrB* variant was acquired in 1 clinical isolate (ARA02-005). Moreover, we identified 36 different variants of *nfxB*, 33 different variants of *mexS*, 3 different variants of *gyrA*, and 1 variant of *gyrB*. Notably, variant NfxBX188C was acquired in 4 genomic backgrounds (6 populations) and it disrupts the stop codon, possibly having an important phenotypic impact. In addition, we observed a variant in which an isoleucine replaces the threonine at position 83 of GyrA (BAL02-001), which is among the most frequent alterations associated with ciprofloxacin resistance in P. aeruginosa occurring in clinical and in *in vitro*-selected resistant mutants (39, 41, 43–45, 56–58), and another in which an alanine replaces the threonine at position 83 of GyrA (CAT02-004), further supporting our experimental approach.

It is important to note that while new mutations were acquired in *nfxB* or *mexS* in 11 or 10 genomic backgrounds, respectively, presenting or not original mutations in genes associated with quinolone resistance, target mutations in *gyrAB* were acquired in only 4 different genomic backgrounds (Fig. 2; Table S3). These results suggest that mutations in regulators of the expression of efflux pumps normally precede target mutations in the acquisition of quinolone resistance, a feature in agreement with findings described for clinical strains isolated from patients treated with ciprofloxacin (59).

In this work we describe a conserved CS to tobramycin or aztreonam in 18 or 16 out of 25 clinical genomic backgrounds subjected to short-term ALE on ciprofloxacin (Fig. 1). This phenotypic convergence toward CS to aminoglycosides has been previously described for ciprofloxacin-resistant clinical isolates of P. aeruginosa (20). In addition, we have recently found that selection of quinolone-resistant mutants is associated with a robust CS to tobramycin in a set of varied preexisting isogenic resistant mutants of P. aeruginosa PA14 (38). Notably, the strength of CS to these antibiotics was considerably higher in the mutants presenting genetic variations in *mexS* or *nfxB*. In agreement with the findings of this work, our results support the notion that mutations in *nfxB* are associated with CS to tobramycin and aztreonam. This is easily deduced from the analysis of the ciprofloxacin-resistant replicate populations belonging to the ARA02-005 (replicates 1, 2, and 3), BAL04-002 (replicates 2, 3, and 4), or CAT09-004 (replicates 2, 3, and 4) genomic background, which only acquired mutations in *nfxB* and whose tobramycin or aztreonam MICs were reduced up to 15.8-, 5.3-, and 6-fold or 2.6-, 2.6-, and 3-fold, respectively, with respect to their parental strains. In addition, mutations in *mexS* are likely responsible for the CS to tobramycin and aztreonam observed in different genomic backgrounds, such as replicate populations from the AND04-004A (all replicates), CAT09-003 (replicates 3 and 4), or CAT06-005 (all replicates) genomic background, which only acquired mutations in this gene and showed a 2.7-, 4-, and 2.7-fold or 2-, 5.3-, and 3-fold reduction of tobramycin or aztreonam MIC, respectively. In addition, we observed that the BAL02-001 isolate (replicate population 2), which acquired genetic variations only in *gyrA*, presented a 2-fold reduction of tobramycin MIC. This feature supports the idea that CS associated with *gyrAB* mutations was weaker than that caused by *nfxB* or *mexS* mutations.

It is worth mentioning that the genes mutated during our ALE experiments in the presence of ciprofloxacin are also the most frequently found mutated in patients infected by P. aeruginosa treated with ciprofloxacin (39, 41, 43–45, 56–59). In agreement with clinical observations, showing that genetic variations in *nfxB* are acquired early during ciprofloxacin treatments (59), mutations in *nfxB* were frequently selected during this work, presenting the greatest decrease of tobramycin (CAT09-004) or aztreonam (ARA02-005 and CAT09-004) MICs (Tables S1 and S3).

Overall, these results provide information on CS robustness associated with the use of ciprofloxacin in bacteria presenting different genomic backgrounds, which is fundamental for its exploitation in clinical settings.

### Robustness of collateral sensitivity may guide evolutionary strategies to drive antibiotic-resistant clinical strains of P. aeruginosa to extinction.

In this work, we have identified a robust pattern of CS to tobramycin or aztreonam in distinct genomic backgrounds of P. aeruginosa, including isolates belonging to high-risk epidemic STs (Fig. 1; Table S1). Given that we have recently described, using preexisting antibiotic-resistant mutants of P. aeruginosa PA14, the potential exploitation of the conservation of CS to both drugs associated with the use of ciprofloxacin (38) and that a previous work supported the efficacy of both aminoglycoside and β-lactam drugs to eradicate quinolone-resistant populations of P. aeruginosa from cystic fibrosis patients (20), we raised the possibility of extrapolating our evolutionary observations to the clinic by studying the effect of such combinations in clinical isolates from different STs and presenting different preexisting resistomes. To that aim, we chose 6 clinical isolates presenting a robust pattern of CS to tobramycin and aztreonam (FQSE11-1010, ARA02-005, BAL04-002, CLE03-006, CAT09-004, and AND04-004A), some of them originally containing ciprofloxacin resistance mutations (Table 1). In addition, among these isolates, two of them belonged to the high-risk epidemic clone ST244 (CAT09-004 and AND04-004A).

**FIG 3:**
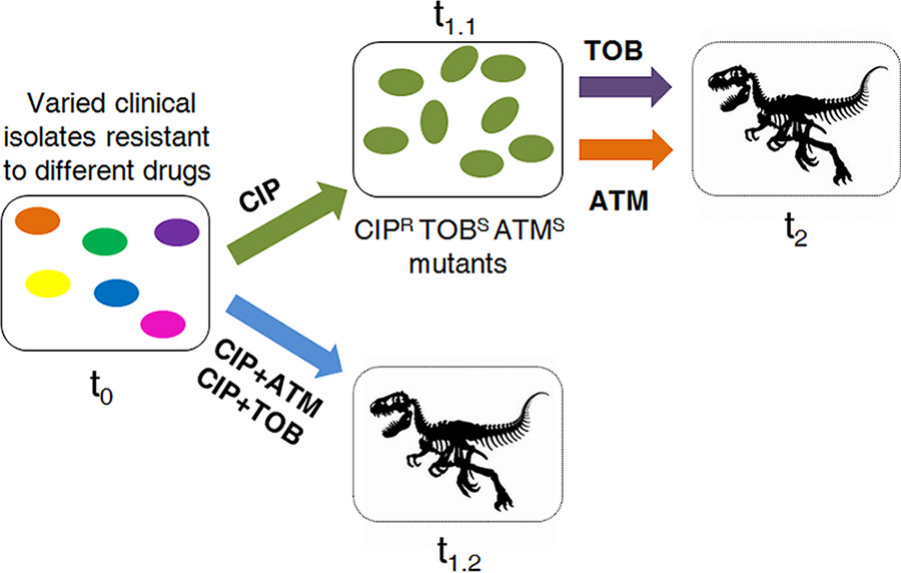
Conceptual figure illustrating the evolution of antibiotic-resistant clinical strains of P. aeruginosa subjected to the alternation of ciprofloxacin with aztreonam or tobramycin or to the combination of ciprofloxacin with tobramycin or ciprofloxacin with aztreonam. Evolution starts when different antibiotic-resistant clinical isolates, represented as cells with different colors, are treated with ciprofloxacin or the combinations ciprofloxacin-tobramycin or ciprofloxacin-aztreonam at time zero (t_0_). In the case of the alternate treatment, there is a first step of evolution toward ciprofloxacin resistance and CS to tobramycin and aztreonam (green cells), rendering ciprofloxacin ineffective (t_1.1_). Then, treatment is switched to tobramycin or aztreonam, which may result in the elimination (represented as a dinosaur fossil skeleton) of cells susceptible to those drugs (t_2_). In the case of the combinatory therapy, evolution consists in a single treatment of the different antibiotic-resistant clinical isolates (t_0_) with ciprofloxacin-tobramycin or ciprofloxacin-aztreonam. Since the acquisition of ciprofloxacin resistance may lead to CS to tobramycin and aztreonam, it could be expected that drug combinations result in a reduced rate of adaptation or the extinction (represented as a dinosaur fossil skeleton) of cells (t_1.2_).

Our first strategy consisted of a first step on ciprofloxacin, to drive evolution toward CS to tobramycin and aztreonam, and a second step on aztreonam or tobramycin, which could drive aztreonam-tobramycin-susceptible cells to extinction (Fig. 3). Between treatments, cells were stocked with glycerol at −80°C to decouple CS from potential negative hysteresis situations (60) that might compromise the interpretation of the results. Since it is known that CS not only improves treatments when antibiotics are applied sequentially but may also optimize combinatory therapy (16), our second strategy consisted of a single step in the presence of the ciprofloxacin-tobramycin or the ciprofloxacin-aztreonam drug pair (Fig. 3).

**FIG 4:**
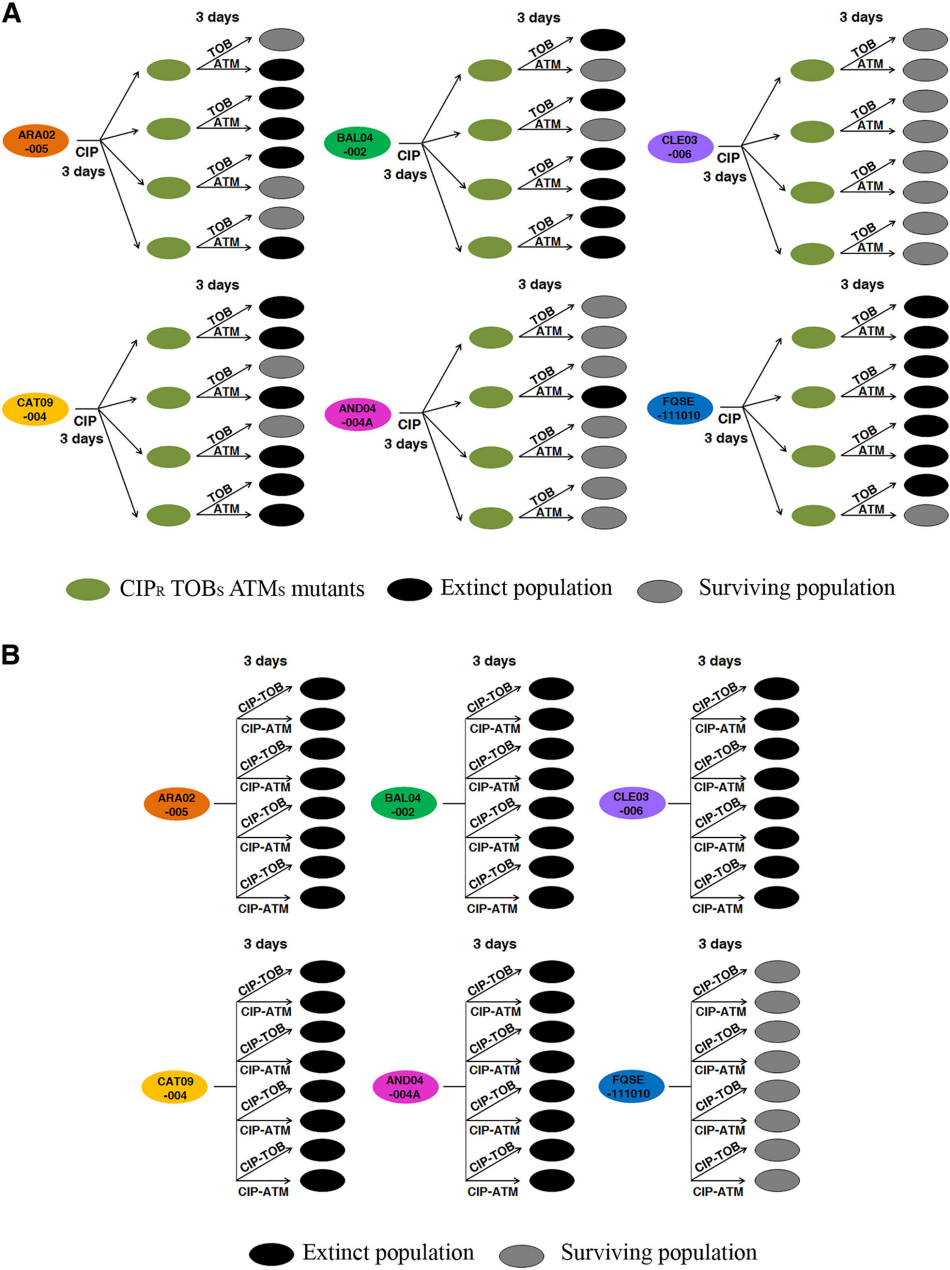
Diagram showing alternation of ciprofloxacin with tobramycin or aztreonam and the combination of ciprofloxacin-tobramycin or ciprofloxacin-aztreonam to drive different antibiotic-resistant clinical strains of P. aeruginosa to extinction. (A) Short-term evolution was performed for 6 clinical isolates (FQSE11-1010, ARA02-005, BAL04-002, CLE03-006, CAT09-004, and AND04-004A), each represented as a cell with a different original color, with 4 replicate populations of each parental strain and for 6 days: 3 days in the presence of ciprofloxacin (or with the absence of antibiotic as a control), leading to ciprofloxacin-resistant populations (green cells), and 3 days in the presence of aztreonam or tobramycin. Extinct populations at the end of the experimental evolution are represented in black, while surviving populations are colored in gray. Thirteen and 12 out of 24 populations subjected to short-term ALE in the presence of tobramycin and aztreonam, respectively, became extinct after 3 days. This evolutionary strategy was ineffective in driving some replicate populations to extinction. (B) Short-term evolution of 6 clinical isolates (FQSE11-1010, ARA02-005, BAL04-002, CLE03-006, CAT09-004, and AND04-004A), represented as a cell with a different original color, with 4 replicate populations of each parental strain, was performed for 3 days in the presence of the ciprofloxacin-aztreonam and the ciprofloxacin-tobramycin combinations. Growth of the 72 control populations was confirmed for the 3 drugs independently used, at the same concentrations used for the drug combinations. Twenty out of 24 populations subjected to short-term ALE in the presence of each antibiotic combination became extinct. In all populations in which extinctions were observed, the differences with respect to the control were statistically significant (*P* < 0.001). These results indicate that CS may not only improve treatment when drugs are applied sequentially but also that this is even more useful to optimize combinatory therapy, including for the high-risk epidemic clones CAT09-004 and AND04-004A.

We started the alternate therapy using the above-described ciprofloxacin-resistant populations, belonging to the different genomic backgrounds, which had been previously subjected to short-term ALE in the presence of ciprofloxacin. Four replicate populations of each, making a total of 24 populations, were studied. In addition, 24 populations not previously challenged with ciprofloxacin were included in the study as controls. The ciprofloxacin-evolved populations presented a ciprofloxacin MIC above the EUCAST clinical breakpoint (0.5 μg/mL) (Table S1) and a reduced aztreonam and tobramycin MIC with respect to their parental strains, being all MICs below the EUCAST clinical breakpoints (16 and 2 μg/mL, respectively), with the exception of tobramycin MIC in the FQSE11-1010 clinical isolate, possibly due to the presence in this strain of the genes *aacA4* and *aadB*, encoding two aminoglycoside-modifying enzymes (Table 1). At this point, we switched selective pressure (Fig. 3) from ciprofloxacin to either aztreonam or tobramycin (24 ciprofloxacin-resistant populations and 24 control populations for each drug). As shown in Fig. 4A, 13 and 12 out of 24 ciprofloxacin-resistant populations subjected to short-term ALE in the presence of aztreonam or tobramycin, respectively, became extinct. Overall, the differences in extinction with respect to the control were statistically significant (*P* < 0.001) for each of the ALE strategies. These results suggest that exploiting CS to aztreonam and tobramycin associated with the use of ciprofloxacin by switching the selective pressure from ciprofloxacin to aztreonam or tobramycin would be ineffective, since this does not drive some replicate populations from 5 and 4 out of 6 clinical isolates, respectively, to extinction at the concentrations tested (see Materials and Methods).

Subsequently, we analyzed the antibiotic combination efficacy of the two drug pairs ciprofloxacin-tobramycin and ciprofloxacin-aztreonam in the 6 original genomic backgrounds. In our previous work, using isogenic resistant mutants, we determined that the high efficacy of these drug combinations is due not to synergistic interactions between ciprofloxacin and tobramycin or aztreonam but to CS associated with acquisition of resistance to ciprofloxacin (38). We subjected 4 replicate populations of each of the 6 different genomic backgrounds to the drug combination ciprofloxacin-aztreonam (24 populations) or ciprofloxacin-tobramycin (24 populations). In addition, we grew 24 populations in each of the 3 antibiotics separately, using the same concentrations as present in each drug pair (72 control populations) (see Materials and Methods). As shown in Fig. 4B, 20 out of 24 populations subjected to short-term ALE in the presence of either ciprofloxacin-tobramycin or ciprofloxacin-aztreonam became extinct, while every control population thrived, as expected. In both cases, the overall differences between the extinction of the populations subjected to selective pressure with respect to the extinction observed in the absence of antibiotic (control populations) were statistically significant (*P* < 0.001). These results indicate that the two antibiotic pairs (ciprofloxacin-tobramycin and ciprofloxacin-aztreonam) are effective in driving the clinical isolates analyzed to extinction, with the exception of FQSE11-1010, which originally presented two aminoglycoside-modifying enzymes and, therefore, an important level of resistance to tobramycin (Table S1). It should be noted that this evolutionary strategy was effective against the high-risk epidemic clones CAT09-004 and AND04-004A (ST244).

Overall, our results support the idea that exploiting CS to tobramycin and aztreonam associated with the use of ciprofloxacin is a possibility that should be considered for the treatment of infections caused by P. aeruginosa, particularly using the combination of ciprofloxacin-tobramycin or ciprofloxacin-aztreonam.

### Conclusions.

A recent retrospective analysis of almost 450,000 antimicrobial susceptibility tests performed during a 4-year period in 23 hospitals from the University of Pittsburgh supports that treatment strategies based on alternating antibiotics may be ineffective at the species level, with exception of a few antibiotic pairs, due to differences in CS patterns at the subpopulation level (61). This means that while novel therapeutic strategies that exploit trade-offs of antibiotic resistance evolution—such as CS—can improve the efficacy of available antibiotics (11), efforts should be made to identify the few cases in which this phenotype is robust and conserved in different genomic backgrounds.

We recently identified a robust pattern of CS to tobramycin and aztreonam that emerges when ciprofloxacin is used against different mutational backgrounds of P. aeruginosa PA14 (38). However, the translation of these results into clinical practice still required them to be confirmed in different genomic backgrounds of P. aeruginosa. In this study, we analyzed CS robustness in 25 clinical strains of P. aeruginosa from different STs, including recognized high-risk epidemic clones belonging to the ST111, ST175, and ST244 (10, 54), with different antibiotic susceptibility profiles and different mutational resistomes. As previously reported (38), although different genetic variations were acquired depending on the original genomic background, they led to a robust pattern of CS to tobramycin and aztreonam. Further, we demonstrated that this trade-off may be exploited to drive different clinical strains of P. aeruginosa to extinction, including the high-risk epidemic clones CAT09-004 and AND04-004A, as well as clinical strains originally presenting ciprofloxacin resistance mutations, such as CLE03-006, by combining ciprofloxacin with either tobramycin or aztreonam. However, it is important to highlight that the combination of ciprofloxacin with tobramycin was unable to drive the FQSE11-1010 isolate to extinction, which originally presented in its genome two acquired aminoglycoside-modifying enzymes. This suggests that the acquisition of antibiotic resistance genes could limit the efficacy of these therapeutic strategies.

We conclude that robust CS patterns could allow the design of new evolutionary strategies to drive bacterial infections to extinction. Although extensive work is still required to apply evolution-based approaches in the clinic, we believe that our results provide sufficient evidence to suggest that the time has come to translate the evolutionary knowledge into medical advances to tackle antibiotic resistance.

## MATERIALS AND METHODS

### Culture conditions and antibiotic susceptibility assays.

Unless otherwise stated, all bacteria were grown in lysogeny broth (LB) (Lennox; Pronadisa) at 37°C with shaking at 250 rpm in glass tubes. MICs of ciprofloxacin, aztreonam, and tobramycin were determined at 37°C, in Mueller-Hinton (MH) agar, using Etest strips (MIC test strip; Liofilchem).

### Short-term adaptive laboratory evolution experiments in the presence of ciprofloxacin.

ALE assays in the presence of ciprofloxacin were performed as described previously (38). Two hundred bacterial populations from stock cultures of 25 P. aeruginosa clinical isolates from different Spanish hospitals (Table 1) were subjected to short-term ALE—4 replicates of each—for 3 days in the presence or absence (control populations) of ciprofloxacin. Cultures were grown at 37°C and 250 rpm in independent glass tubes to avoid cross-contamination. Every day, the cultures were diluted (1/100), adding 10 μL of bacteria to 1 mL of fresh LB containing the concentration of ciprofloxacin (close to MIC) that hindered—but allowed—the growth of each P. aeruginosa isolate under these culture conditions (0.01 μg/mL for BAL02-001; 0.025 μg/mL for CAN01-002 and PAMB148; 0.05 μg/mL for FQSE10-0503, ICA01-004, and GAL02-002; 0.075 μg/mL for CLE03-004, CAT09-003, and CVA02-003; 0.1 μg/mL for NAV01-005, FQSE11-1010, BAL02-009, BAL04-002, AND04-004A, and MAD04-002; 0.2 μg/mL for CAT09-004, FQSE11-0603, FQSE15-0803, MAD05-008, and CAT02-004; 0.3 μg/mL for ARA02-005; 0.5 μg/mL for FQSE24-0304; 0.8 μg/mL for ARA03-004; 2 μg/mL for CLE03-006; and 10 μg/mL for CAT06-005), this concentration being half of the MIC of each parental strain or greater, or without antibiotic (control populations). During the 3 days, the concentration of ciprofloxacin was maintained. Every replicate population was preserved at −80°C at the end of the ALE. In addition, MICs of the antibiotic used for selection and of tobramycin and aztreonam were determined as previously described.

### Determination of subpopulations’ abundance presenting different levels of resistance within evolved populations.

One hundred microliters from a 10^−6^ dilution of an overnight culture of evolved populations presenting a mixture of subpopulations with different antibiotic resistance levels was plated in MH agar plates containing different concentrations of the respective antibiotic. Ciprofloxacin concentrations ranged from 0.015 μg/mL to 0.5 μg/mL, aztreonam concentrations ranged from 0.0015 μg/mL to 0.05 μg/mL, and tobramycin concentrations ranged from 0.03 μg/mL to 16 μg/mL. After 24 h at 37°C, the colonies grown in each antibiotic concentration were counted, and a concentration at which the susceptible subpopulation did not grow but the resistant subpopulation did was used as a threshold to discern between the two populations. The percentage of bacteria belonging to the resistant subpopulation was calculated by dividing the number of colonies grown in the highest antibiotic concentration—where the resistant subpopulation grew—by the number of colonies that grew in the absence of antibiotic. The values were determined as the average of three independent replicates for each strain and condition.

### Alternation of ciprofloxacin with aztreonam or tobramycin.

Short-term ALE experiments in the presence of aztreonam or tobramycin were performed for 3 days, at 37°C and 250 rpm in glass tubes, in 24 populations previously challenged with ciprofloxacin for 3 days and belonging to 6 different clinical isolates (FQSE11-1010, ARA02-005, BAL04-002, CAT09-004, AND04-004A, and CLE03-006). These 24 ciprofloxacin-resistant populations and 24 control populations (not challenged with ciprofloxacin) were grown from glycerol stocks, and every day, for 3 days, the cultures were diluted (1/100) in fresh LB containing aztreonam or tobramycin at the concentration (close to MIC) that hindered—but allowed—the growth of each parental P. aeruginosa isolate under these culture conditions (tobramycin, 0.75 μg/mL for ARA02-005, CAT09-004, and AND04-004A, 1 μg/mL for BAL04-002, and 512 μg/mL for FQSE11-1010; aztreonam, 1.5 μg/mL for FQSE11-1010, 3 μg/mL for ARA02-005, 5 μg/mL for BAL04-002, CAT09-004, and CLE03-006; and 6 μg/mL for AND04-004A), this concentration being half of the MIC of each parental strain or greater. Extinction of the populations was determined by measuring the absorbance (optical density at 600 nm [OD_600_]) of 100 μL of bacterial cultures at the end of the ALE assay, in a 96-well microtiter plate (Nunc) in a Tecan Infinite 200 plate reader, and by plating out 50 μL of final cultures on LB agar to look for viable cells. The absence of colonies was interpreted as extinction of the population.

### Combination of ciprofloxacin with tobramycin or aztreonam.

Four replicate populations from 6 different isolates (FQSE11-1010, ARA02-005, BAL04-002, CAT09-004, AND04-004A, and CLE03-006) were grown from glycerol stocks. Every day for 3 days, the cultures were diluted (1/100) in fresh LB medium containing a ciprofloxacin-tobramycin combination (24 populations), a ciprofloxacin-aztreonam combination (24 populations), or each single drug (72 control populations). Each single antibiotic was added at the concentration (close to MIC) that hindered—but allowed—the growth of each parental isolate of P. aeruginosa under these culture conditions. Extinction of the populations was determined by measuring the absorbance (OD_600_) of 100 μL of bacterial culture at the end of the ALE assay, in a Tecan Infinite 200 plate reader using a 96-well microtiter plate (Nunc), and by plating out final cultures on LB agar to look for viable cells.

### Whole-genome sequencing and analysis of genetic changes.

The genomic DNA extraction and DNA quality analysis of the 63 selected ciprofloxacin-resistant populations were performed by the Translational Genomics Unit (Instituto Ramón y Cajal de Investigación Sanitaria—Hospital Ramón y Cajal from Madrid). Genomic DNA of each population was extracted by Chemagic DNA bacterial kit H96 (CMG-799 Chemagic) using the Chemagic 360/MSMI instrument (PerkinElmer). The assay of DNA quality was performed using an Agilent 2200 TapeStation system. Library construction and whole-genome sequencing were performed by the Oxford Genomics Centre. Pair-end libraries (2 × 150) were sequenced using an Illumina NovaSeq6000 system. Coverage was higher than 150× for all samples.

Obtained paired-end reads were mapped to the P. aeruginosa PAO1 reference genome (GenBank accession number NC_002516.2) with Bowtie 2 v2.2.4 and pileup and raw files were obtained by using SAMtools v0.1.16 and PicardTools v1.140, using the Genome Analysis Toolkit (GATK) v3.4.46 for realignment around indels (9). Single-nucleotide polymorphisms (SNPs) and microindels were investigated in a set of chromosomal genes known to be involved in P. aeruginosa antibiotic resistance (*gyrB*, *mexR*, *mexA*, *mexB*, *oprM*, *ampDh3*, *parS*, *parR*, *mexY*, *mexX*, *mexZ*, *galU*, *mexS*, *mexT*, *mexE*, *mexF*, *oprN*, *dacB*, *gyrA*, *nalD*, *nalC*, *dacC*, *pbpA*, *mpl*, *ampR*, *ampC*, *fusA1*, *ftsI*, *ampD*, *oprJ*, *mexD*, *mexC*, *nfxB*, *pmrA*, *pmrB*, *parC*, *parE*, *armZ*, and *ampDh2*) (62) and were extracted from the raw and the totalpileup files if at least 100 reads covered such positions and if the change was present in at least 10% of the reads. SNPs and MICROINDELS were annotated with SnpEff v4.2. Additionally, paired-end reads were *de novo* assembled using SPAdes v3.13.1 and gene absence was evaluated using the SeqMonk program. Likewise, OprD structural integrity was investigated using an appropriate reference sequence. Finally, all mutations already present in the parental strain were filtered.

The presence of horizontally acquired antimicrobial resistance determinants was also investigated using the web tool ResFinder (https://cge.cbs.dtu.dk/services/ResFinder/) and the ST was determined using the web tool MLST 2.0 (https://cge.cbs.dtu.dk/services/MLST/).

### Statistical analysis.

MIC data were first analyzed for normality using the Shapiro-Wilk test. Most data were not normal and were analyzed using the nonparametric asymptotic Wilcoxon-Mann-Whitney U test calculated with the Streitberg-Röhmel and van de Wiel algorithms, as implemented in R package “coin,” directly on raw data to avoid imprecisions due to ties. To assert the presence of a substantively significant effect size, we computed the fold change and log transformed it to obtain log_2_ fold change. Log_2_ fold change was checked for normality with the Shapiro-Wilk test and then compared against μ of 1 for significance using a single-sample *t* test. Comparisons of log_2_ fold change between control ALE-subjected strains were done using a bilateral *t* test. Constant data were detected prior to *t* test calculation and handled manually. All comparison tests (parametric and nonparametric) were additionally corrected with Benjamini-Hochberg correction for multiple comparisons to avoid biases due to the high number of strains considered. Analysis was performed first on the ensemble of all strains subjected to a given treatment and then, as all tests were significant, on observations of each strain separately. In each case, a significant comparison test was followed by a unilateral test to confirm the direction of change. When evolved populations contained different subpopulations presenting diverse MICs, the MIC with a greater change regarding the parental strain was used for statistical analysis.

The significance of the variation in the number of extinct populations in the alternating and combinatory ALE experiments was estimated using McNemar’s and the log-likelihood ratio test (G-test) as implemented in R package “Deducer.” All statistical analyses were performed using the R package (http://cran.r-project.org).

### Data availability.

Sequence files of derivatives obtained under ciprofloxacin exposure have been deposited in the European Nucleotide Archive under study number PRJEB56068. The genomic sequences of the original strains can be found in under BioProject numbers PRJEB40140, PRJEB19788, and PRJEB24151. The rest of the data needed to evaluate the conclusions in the paper are present in the paper and/or the Supplemental Material.
