# Supplementary material for: Rapid Phenotypic Convergence towards Collateral Sensitivity in Clinical Isolates of Pseudomonas aeruginosa Presenting Different Genomic Backgrounds
Source: Microbiol Spectr. 2022 Dec 19;11(1):e02276-22. doi: 10.1128/spectrum.02276-22 (PMC9927454; doi:10.1128/spectrum.02276-22)
Supplement: Supplemental file 1 — Supplemental material. Download spectrum.02276-22-s0001.pdf, PDF file, 0.3 MB [file spectrum.02276-22-s0001.pdf]

Supplementary Table 1. Susceptibility to ciprofloxacin, aztreonam and tobramycin of the final populations of *Pseudomonas aeruginosa* after ALE on ciprofloxacin.

Population	MIC (µg/ml)		
	CIP	ATM	TOB
NAV01-005	0.5	0.38	12
NAV01-005 CIP 1	6	0.25	8
NAV01-005 CIP 2	6	0.19	8/3* (42%)
NAV01-005 CIP 3	4	0.19	6/2 (14%)
NAV01-005 CIP 4	6	0.19	12/4 (37%)
ARA03-004	1	8	4
ARA03-004 CIP 1	12	4	2/1 (14%)
ARA03-004 CIP 2	12	64	3
ARA03-004 CIP 3	8	2	1
ARA03-004 CIP 4	8	8	3
CVA02-003	0.38	4	48
CVA02-003 CIP 1	0.75	3	48
CVA02-003 CIP 2	0.75	4	32
CVA02-003 CIP 3	0.75	4	48
CVA02-003 CIP 4	0.75	4	32
FQSE111010	0.5	0.75	≥256**
FQSE111010 CIP 1	6	0.38/0.19 (37%)	128
FQSE111010 CIP 2	4	0.38/0.19 (30%)	128
FQSE111010 CIP 3	4	0.38/0.19 (43%)	128
FQSE111010 CIP 4	4	0.38/0.25 (44%)	192
ARA02-005	0.125	3	1
ARA02-005 CIP 1	4	0.19	0.38
ARA02-005 CIP 2	3	0.25	0.38
ARA02-005 CIP 3	4	0.25	0.38

ARA02-005 CIP 4	12	0.38	0.25
BAL02-009	1	2	0.5
BAL02-009 CIP 1	4	1.5	0.5
BAL02-009 CIP 2	6	1.5	0.5
BAL02-009 CIP 3	16	1.5	0.5
BAL02-009 CIP 4	6	1.5	0.5
BAL04-002	0.125	4	1
BAL04-002 CIP 1	1.5	4/0.75 (43%)	0.5
BAL04-002 CIP 2	2	0.75	0.38
BAL04-002 CIP 3	1.5	1	0.5
BAL04-002 CIP 4	2	0.75	0.5
CAN01-002	0.032	0.38	2
CAN01-002 CIP 1	1.5	0.19	0.75
CAN01-002 CIP 2	1	0.38	4
CAN01-002 CIP 3	1	0.19	1
CAN01-002 CIP 4	0.5	0.25	0.75
CAT09-004	0.094	3	1.5
CAT09-004 CIP 1	3	0.5	0.25
CAT09-004 CIP 2	4	0.5	0.5
CAT09-004 CIP 3	1.5	0.75	0.5
CAT09-004 CIP 4	12	0.5	0.5
FQSE110603	0.125	0.38	3
FQSE110603 CIP 1	2	0.25	1
FQSE110603 CIP 2	2	0.25	1
FQSE110603 CIP 3	2	0.25	0.75
FQSE110603 CIP 4	3	0.25	1
AND04-004A	0.125	4	1
AND04-004A CIP 1	1	1	0.5
AND04-004A CIP 2	1	1.5	0.5

AND04-004A CIP 3	0.75/12 (34%)	1.5	0.5
AND04-004A CIP 4	0.75	2	0.5
BAL02-001	0.047	6	2
BAL02-001 CIP 1	0.19	48	2
BAL02-001 CIP 2	0.38	16/6 (82%)	1
BAL02-001 CIP 3	0.19	48	1.5
BAL02-001 CIP 4	0.25	48	2
CAT06-005	16	4	48
CAT06-005 1	≥32**	1.5	24
CAT06-005 CIP 2	≥32**	3	24
CAT06-005 CIP 3	≥32**	2	24
CAT06-005 CIP 4	≥32**	3	16
FQSE15-0803	0.38	0.38	3
FQSE15-0803 CIP 1	2/6 (16%)	0.25	1.5
FQSE15-0803 CIP 2	3	0.38	1
FQSE15-0803 CIP 3	1.5/16 (25%)	0.38/1 (46%)	2
FQSE15-0803 CIP 4	2	0.38	2
FQSE24-0304	3	0.38	4
FQSE24-0304 CIP 1	≥32**	0.75	4
FQSE24-0304 CIP 2	≥32**	0.5	3
FQSE24-0304 CIP 3	≥32**	0.38	4
FQSE24-0304 CIP 4	≥32**	0.5	3
CAT02-004	0.5	12	0.75
CAT02-004 CIP 1	6	12	0.38
CAT02-004 CIP 2	2	6	0.38
CAT02-004 CIP 3	2	8	0.75
CAT02-004 CIP 4	3	6	0.38
CAT09-003	0.25	6	≥256**
CAT09-003 CIP 1	2	0.75	24

CAT09-003 CIP 2	1.5	4	≥256**
CAT09-003 CIP 3	1	3	≥256**
CAT09-003 CIP 4	1.5	1.5	48
CLE03-004	0.125	3	1
CLE03-004 CIP 1	2	2	0.75
CLE03-004 CIP 2	2	1.5	0.75
CLE03-004 CIP 3	2	1.5	0.75
CLE03-004 CIP 4	2	0.75	1
CLE03-006	3	4	4
CLE03-006 CIP 1	≥32**	1.5	1.5
CLE03-006 CIP 2	≥32**	1	1.5
CLE03-006 CIP 3	≥32**	1	1.5
CLE03-006 CIP 4	≥32**	0.75	1.5
GAL02-002	0.38	8	8
GAL02-002 CIP 1	1	8	6
GAL02-002 CIP 2	0.75	6	8
GAL02-002 CIP 3	0.75	12	8
GAL02-002 CIP 4	1	8	8
ICA01-004	0.094	4	1.5
ICA01-004 CIP 1	1.5	1.5	0.75
ICA01-004 CIP 2	2	16	1.5
ICA01-004 CIP 3	1	2	2
ICA01-004 CIP 4	0.75	2	1
MAD04-002	0.19	6	2
MAD04-002 CIP 1	3	3	1
MAD04-002 CIP 2	3	2	1
MAD04-002 CIP 3	3	6	1
MAD04-002 CIP 4	3	4	1
MAD05-008	0.5	8	4

MAD05-008 CIP 1	4	2	2
MAD05-008 CIP 2	3	2	3
MAD05-008 CIP 3	2	2	2
MAD05-008 CIP 4	3	2	2
PAMB148	0.094	96	4
PAMB148 CIP 1	1.5	96	3
PAMB148 CIP 2	1	96	3
PAMB148 CIP 3	1	128	3
PAMB148 CIP 4	0.75	128	3
FQSE10-0503	0.5	3	4
FQSE10-0503 CIP 1	4	4	3
FQSE10-0503 CIP 2	3	3	3
FQSE10-0503 CIP 3	3	4	2
FQSE10-0503 CIP 4	2	16	6

CIP: ciprofloxacin, ATM: aztreonam, TOB: tobramycin.

* MICs of mixed populations are indicated as two different values separated by a forward slash (the percentage of bacteria belonging to the more resistant subpopulation is indicated in parenthesis).

** MICs were above limits of detection.

EUCAST breakpoints for CIP, ATM and TOB are 0.5, 16 and 2 µg/ml, respectively.

Supplementary Table 2. Ciprofloxacin, aztreonam and tobramycin MIC values (µg/ml) in the final control populations of *P. aeruginosa* after ALE with no antibiotic.

Population	MIC (µg/ml)		
	CIP	ATM	TOB
NAV01-005	0.5	0.38	12
NAV01-005 Control 1	0.38	0.38	4
NAV01-005 Control 2	0.38	0.38	6
NAV01-005 Control 3	0.38	0.38	6
NAV01-005 Control 4	0.5	0.38	8
ARA03-004	1	8	4
ARA03-004 Control 1	1	12	4
ARA03-004 Control 2	1	12	4
ARA03-004 Control 3	0.75	12	4
ARA03-004 Control 4	1	12	3
CVA02-003	0.38	4	48
CVA02-003 Control 1	0.5	4	48
CVA02-003 Control 2	0.5	4	48
CVA02-003 Control 3	0.5	4	32
CVA02-003 Control 4	0.5	4	48
FQSE111010	0.5	0.75	≥256*
FQSE111010 Control 1	0.5	0.5	≥256*
FQSE111010 Control 2	0.5	0.75	≥256*
FQSE111010 Control 3	0.5	0.5	≥256*
FQSE111010 Control 4	0.5	0.75	≥256*
ARA02-005	0.125	3	1
ARA02-005 Control 1	0.094	4	1
ARA02-005 Control 2	0.125	3	1.5
ARA02-005 Control 3	0.094	3	1
ARA02-005 Control 4	0.125	3	1

BAL02-009	1	2	0.5
BAL02-009 Control 1	1	2	0.5
BAL02-009 Control 2	1	2	0.5
BAL02-009 Control 3	1.5	2	0.5
BAL02-009 Control 4	1	1.5	0.38
BAL04-002	0.125	4	1
BAL04-002 Control 1	0.094	3	1
BAL04-002 Control 2	0.094	3	1.5
BAL04-002 Control 3	0.064	3	1.5
BAL04-002 Control 4	0.125	3	1
CAN01-002	0.032	0.38	2
CAN01-002 Control 1	0.38	0.38	1.5
CAN01-002 Control 2	0.19	0.25	1.5
CAN01-002 Control 3	0.38	0.25	1.5
CAN01-002 Control 4	0.25	0.25	2
CAT09-004	0.094	3	1.5
CAT09-004 Control 1	0.064	2	1.5
CAT09-004 Control 2	0.094	3	1.5
CAT09-004 Control 3	0.094	3	1.5
CAT09-004 Control 4	0.094	3	1.5
FQSE110603	0.125	0.38	3
FQSE110603 Control 1	0.125	0.38	3
FQSE110603 Control 2	0.125	0.38	3
FQSE110603 Control 3	0.125	0.38	3
FQSE110603 Control 4	0.094	0.38	2
AND04-004A	0.125	4	1
AND04-004A Control 1	0.094	6	1
AND04-004A Control 2	0.064	6	1
AND04-004A Control 3	0.094	4	1.5

AND04-004A Control 4	0.094	4	1.5
BAL02-001	0.047	6	2
BAL02-001 Control 1	0.047	6	2
BAL02-001 Control 2	0.047	6	1.5
BAL02-001 Control 3	0.047	8	2
BAL02-001 Control 4	0.047	6	2
CAT06-005	16	4	48
CAT06-005 Control 1	16	4	48
CAT06-005 Control 2	16	4	48
CAT06-005 Control 3	16	4	48
CAT06-005 Control 4	16	4	32
FQSE15-0803	0.38	0.38	3
FQSE15-0803 Control 1	0.25	0.38	3
FQSE15-0803 Control 2	0.25	0.38	3
FQSE15-0803 Control 3	0.25	0.38	3
FQSE15-0803 Control 4	0.25	0.38	3
FQSE24-0304	3	0.38	4
FQSE24-0304 Control 1	3	0.5	4
FQSE24-0304 Control 2	2	0.5	3
FQSE24-0304 Control 3	3	0.38	3
FQSE24-0304 Control 4	3	0.38	4
CAT02-004	0.5	12	0.75
CAT02-004 Control 1	0.5	16	0.75
CAT02-004 Control 2	0.38	12	0.5
CAT02-004 Control 3	0.38	16	0.5
CAT02-004 Control 4	0.5	12	0.5
CAT09-003	0.25	6	≥256*
CAT09-003 Control 1	0.25	6	≥256*
CAT09-003 Control 2	0.25	6	≥256*

CAT09-003 Control 3	0.25	4	≥256*
CAT09-003 Control 4	0.25	6	≥256*
CLE03-004	0.125	3	1
CLE03-004 Control 1	0.19	3	1.5
CLE03-004 Control 2	0.19	3	1
CLE03-004 Control 3	0.125	3	1.5
CLE03-004 Control 4	0.19	3	2
CLE03-006	3	4	4
CLE03-006 Control 1	3	3	4
CLE03-006 Control 2	3	4	4
CLE03-006 Control 3	3	4	4
CLE03-006 Control 4	3	3	3
GAL02-002	0.38	8	8
GAL02-002 Control 1	0.5	8	6
GAL02-002 Control 2	0.5	8	6
GAL02-002 Control 3	0.5	8	8
GAL02-002 Control 4	0.5	8	8
ICA01-004	0.094	4	1.5
ICA01-004 Control 1	0.094	3	3
ICA01-004 Control 2	0.094	3	1.5
ICA01-004 Control 3	0.125	4	2
ICA01-004 Control 4	0.125	3	1.5
MAD04-002	0.19	6	2
MAD04-002 Control 1	0.19	8	3
MAD04-002 Control 2	0.19	6	2
MAD04-002 Control 3	0.19	8	2
MAD04-002 Control 4	0.19	6	2
MAD05-008	0.5	8	4
MAD05-008 Control 1	0.5	6	4

MAD05-008 Control 2	0.5	8	6
MAD05-008 Control 3	0.5	8	8
MAD05-008 Control 4	0.5	8	4
PAMB148	0.094	96	4
PAMB148 Control 1	0.094	96	6
PAMB148 Control 2	0.125	96	6
PAMB148 Control 3	0.125	96	3
PAMB148 Control 4	0.125	96	4
FQSE10-0503	0.5	3	4
FQSE10-0503 Control 1	0.5	4	4
FQSE10-0503 Control 2	0.5	3	3
FQSE10-0503 Control 3	0.5	4	4
FQSE10-0503 Control 4	0.5	4	4

CIP: ciprofloxacin, ATM: aztreonam, TOB: tobramycin

* MICs were above limits of detection.

Supplementary Table 3. Ciprofloxacin, aztreonam and tobramycin MIC values (µg/ml), original resistome and acquired mutations encountered in the final populations of *P. aeruginosa* showing a significant collateral sensitivity after ALE on ciprofloxacin.

ISOLATE ID	ST	Hypermutator	CIP	ATM	TOB	Baseline resistome	Acquired mutations
AND04-004A	244	NO	0.125	4	1		-
AND04-004A CIP 1			1	1	0.5		*mexS* (nt649Δ3-14.6%, nt421Δ1-18%)
AND04-004A CIP 2			1	1.5	0.5		*mexS* (nt240Δ4-19.7%, nt370Δ3-13.6%)
AND04-004A CIP 3			0.75/12*	1.5	0.5		*mexS* (nt649Δ3-15%, nt421Δ1-11.5%)
AND04-004A CIP 4			0.75	2	0.5		*mexS* (nt649Δ3-13.7%, nt421Δ1-26.4%)
ARA02-005	267	NO	0.125	3	1	*oprN* (R151L), *armZ* (R320C)	-
ARA02-005 CIP 1			4	0.19	0.38		*nfxB* (nt46ins9-12.8%, nt60Δ2-12.5%)
ARA02-005 CIP 2			3	0.25	0.38		*nfxB* (nt345Δ1-97.4%)
ARA02-005 CIP 3			4	0.25	0.38		*nfxB* (L14R-100%)
ARA02-005 CIP 4			12	0.38	0.25		*nfxB* (K37M-95.1%, nt113Δ2-18.5%, nt121ins3-11.8%), *gyrB* (aa467Δ1-89.1%)
ARA03-004	845	NO	1	8	4	*gyrA* (D87N), *ampD* (Q131X), *mexT* (G246S), *mexB* (nt54Δ1), *mexZ* (nt302Δ9), *dacB* (nt781Δ1)	-
ARA03-004 CIP 1			12	4	2/1		*nfxB* (nt222Δ1-93.4%)
ARA03-004 CIP 3			8	2	1		*nfxB* (nt108Δ1-98.1%)
BAL02-001	1619	NO	0.047	6	2		-
BAL02-001 CIP 2			0.38	16/6	1		*gyrA* (T83I-90.1%)
BAL04-002	1816	NO	0.125	4	1	*mexA* (K86E), *ampR* (G295R), *ampC* (A278G), *parE* (E215Q)	-
BAL04-002 CIP 1			1.5	4/0.75	0.5		*mexS* (G136D-33%, S41R-39.9%, nt749Δ9-15.9%)
BAL04-002 CIP 2			2	0.75	0.38		*nfxB* (nt61Δ1-91.5%)
BAL04-002 CIP 3			1.5	1	0.5		*nfxB* (A158E-97.8%)
BAL04-002 CIP 4			2	0.75	0.5		*nfxB* (Y118X-98.9%)
CAN01-002	111	NO	0.032	0.38	2		-

CAN01-002 CIP 1			1.5	0.19	0.75	*mexB* (Q319X), *mexY* (G530S), *mexZ* (Q140K), *mexT* (G276D), *oprD* (nt174Δ11), *mexS* (nt300IS)	*nfxB* (aa16ins3-13.3%, nt391Δ19-56%)
CAN01-002 CIP 3			1	0.19	1		*nfxB* (X188C-99%)
CAN01-002 CIP 4			0.5	0.25	0.75		*nfxB* (X188C-94.3%)
CAT02-004	244	NO	0.5	12	0.75	*mexR* (nt266Ins1), *dacB* (A340G)	-
CAT02-004 CIP 1			6	12	0.38		*gyrA* (T83A-91.2%), *mexS* (nt964Δ1-96.8%)
CAT02-004 CIP 2			2	6	0.38		*mexS* (nt964Δ1-61.7%, nt659ins9-21.7%)
CAT02-004 CIP 4			3	6	0.38		*mexS* (nt964Δ1-100%)
CAT06-005	175	NO	16	4	48	*aadB, oprM* (T198P), *oprD* (Q142X), *mexZ* (G195D), *gyrA* (T83I, D87N), *ampR* (G154R), *parC* (S87W), *armZ* (V266M)	-
CAT06-005 CIP 1			≥32	1.5	24		*mexS* (V215F-38.4%)
CAT06-005 CIP 2			≥32	3	24		*mexS* (V215F-32.1%, nt224Δ12-11.1%)
CAT06-005 CIP 3			≥32	2	24		*mexS* (S234P-40.6%, nt532ins1-12.3%)
CAT06-005 CIP 4			≥32	3	16		*mexS* (V215F-30.9%, nt281Δ1-14.1%)
CAT09-003	253	NO	0.25	6	≥256	*aacA4, mexZ* (H51D)	-
CAT09-003 CIP 1			2	0.75	24		Not detected
CAT09-003 CIP 3			1	3	≥256**		*mexS* (L263Q-38.9%, Q200X-51.1%)
CAT09-003 CIP 4			1.5	1.5	48		*mexS* (Q55X-15.6%)
CAT09-004	244	NO	0.094	3	1.5		-
CAT09-004 CIP 1			3	0.5	0.25		*nfxB* (L122R-96.5%), *gyrA* (A51V-34.3%)
CAT09-004 CIP 2			4	0.5	0.5		*nfxB* (Q64X-97.1%)
CAT09-004 CIP 3			1.5	0.75	0.5		*nfxB* (nt499Δ1-94.4%)
CAT09-004 CIP 4			12	0.5	0.5		*nfxB* (L122R-100%)
CLE03-004	381	NO	0.125	3	1	*mexZ* (nt386Δ1)	-
CLE03-004 CIP 2			2	1.5	0.75		*mexS* (V288E-18.9%, P254R-44.6%)
CLE03-004 CIP 3			2	1.5	0.75		*mexS* (nt589ins3-11.4%, nt275Δ1-12.7%)
CLE03-004 CIP 4			2	0.75	1		*mexS* (nt589ins3-26.1%, nt275Δ1-13.7%)
CLE03-006	1337	NO	3	4	4	*parR* (aa214Δ1), *gyrA* (D87N), *ampD* (D59E)	-

CLE03-006 CIP 1			≥32	1.5	1.5		Not detected
CLE03-006 CIP 2			≥32	1	1.5		*nfxB* (nt4Δ1-12.3%)
CLE03-006 CIP 3			≥32	1	1.5		Not detected
CLE03-006 CIP 4			≥32	0.75	1.5		*nfxB* (R42H-20.5%, X188C-59.3%)
ICA01-004	698	NO	0.094	4	1.5	*oprD* (D43N), *mexE* (V156A), *ampD* (E162Q), *armZ* (V266M), *mexY* (D428N)	-
ICA01-004 CIP 1			1.5	1.5	0.75		Not detected
ICA01-004 CIP 3			1	2	2		*mexS* (Q297X-28.5%, A141D-42.9%)
ICA01-004 CIP 4			0.75	2	1		*nfxB* (X188C-93.6%)
FQSE11-0603	701	NO	0.125	0.38	3	*mexB* (nt775Δ1), *mexY* (N709H,A586T), *mexX* (A38P), *oprN* (R363H), *ampDh2* (P116S)	-
FQSE11-0603 CIP 1			2	0.25	1		*mexS* (G63C-68%)
FQSE11-0603 CIP 2			2	0.25	1		*mexS* (nt677ins1-42.5%, nt331Δ1-30.8%)
FQSE11-0603 CIP 3			2	0.25	0.75		*mexS* (nt396ins2-11.5%, nt386Δ2-13.2%, nt316ins10-11.8%, nt300Δ94)
FQSE11-0603 CIP 4			3	0.25	1		*mexS* (nt618Δ1-81.8%)
FQSE11-1010	701	NO	0.5	0.75	≥256	*aacA4, aadB, gyrB* (R138L), *mexY* (N709H, A586T), *mexX* (A38P), *oprN* (R363H), *gyrA* (Y267N), *nfxB* (E75K), *ampDh2* (P116S), *mpl* (Q248X), *mexA* (nt45Δ1)	-
FQSE11-1010 CIP 1			6	0.38/0.19	128		Not detected
FQSE11-1010 CIP 2			4	0.38/0.19	128		Not detected
FQSE11-1010 CIP 3			4	0.38/0.19	128		Not detected
FQSE11-1010 CIP 4			4	0.38/0.25	192		Not detected
FQSE15-0803	274	YES (*mutS*-nt814Δ4)	0.38	0.38	3	*mexA* (L338P), *mexZ* (A144V)	-
FQSE15-0803 CIP 1			2/6	0.25	1.5		*mexS* (nt833ins15-15.4%)
FQSE15-0803 CIP 2			3	0.38	1		*mexS* (nt770ins2-43.7%), *nfxB* (nt102ins5-47%)
MAD04-002	242	NO	0.19	6	2	*parC* (K726R)	-
MAD04-002 CIP 1			3	3	1		*nfxB* (X188C-34.5%, nt55Δ1-14.6%)
MAD04-002 CIP 2			3	2	1		*nfxB* (F177S-38.2%)
MAD04-002 CIP 3			3	6	1		*nfxB* (X188C-45.4%, nt532ins1-11.4%)
MAD04-002 CIP 4			3	4	1		*nfxB* (F177S-37.3%, nt532ins1-21.8%)

MAD05-008	1717	NO	0.5	8	4	*parS* (V152A), *mexY* (T238I)	-
MAD05-008 CIP 1			4	2	2		*nfxB* (R42H-51.8%, nt487Δ1-20.7%)
MAD05-008 CIP 2			3	2	3		*nfxB* (G129X-42.7%, nt315Δ11-23.2%)
MAD05-008 CIP 3			2	2	2		*nfxB* (G58X-100%)
MAD05-008 CIP 4			3	2	2		*nfxB* (P22L-70%)
NAV01-005	1637	NO	0.5	0.38	12	*gyrB* (P749S), *mexB* (P190L), *oprD* (nt742Δ1), *mexZ* (R104W), *galU* (F248S), *fusA1* (Y552C), *mexD* (L1027V), *mexC* (V367A), *pmrB* (A173T), *armZ* (A262S)	-
NAV01-005 CIP 2			6	0.19	8/3		*nfxB* (nt172Δ2-47.4%, nt84Δ17-17.5%), *mexS* (nt61Δ14-10.2%)
NAV01-005 CIP 3			4	0.19	6/2		*nfxB* (nt350Δ11-92.6%)
NAV01-005 CIP 4			6	0.19	12/4		*nfxB* (nt289Δ16-31.7%, nt19ins3-11.8%, nt30Δ1-10.7%)

CIP: ciprofloxacin, ATM: aztreonam, TOB: tobramycin, Δ: deletion, Ins: insertion. IS: insertion sequence. X: stop codon.

* MICs of mixed populations are indicated as two different values separated by a forward slash.
